# Physician-Perceived Barriers to Treating Opioid Use Disorder in the Emergency Department

**DOI:** 10.7759/cureus.19923

**Published:** 2021-11-26

**Authors:** Gideon Logan, Amber Mirajkar, Jessica Houck, Fernando Rivera-Alvarez, Emily Drone, Parth Patel, Alexandra Craen, Larissa Dub, Nubaha Elahi, David Lebowitz, Ayanna Walker, Latha Ganti

**Affiliations:** 1 Emergency Medicine, University of Central Florida/Hospital Corporation of America Graduate Medical Education (HCA GME) Consortium Emergency Medicine Residency Program of Greater Orlando, Orlando, USA; 2 Emergency Medicine, Osceola Regional Medical Center, Kissimmee, USA; 3 Emergency Medicine, Envision Physician Services, Plantation, USA; 4 Emergency Medicine, University of Kentucky College of Medicine, Lexington, USA; 5 Department of Pediatric Emergency Medicine, Orlando Health, Orlando, USA; 6 Emergency Medicine, University of Central Florida College of Medicine, Orlando, USA; 7 Clinical Sciences, University of Central Florida College of Medicine, Orlando, USA; 8 Emergency Medicine, University of Central Florida College of Medicine/Hospital Corporation of America (HCA) Healthcare Graduate Medical Education Consortium of Greater Orlando, Orlando, USA

**Keywords:** opioid epidemic, naltrexone, buprenorphine, methadone, opioid, x-waiver, medication-assisted treatment, opioid use disorder

## Abstract

Objective

We aimed to assess physicians’ perceptions of barriers to starting medication-assisted treatment (MAT) in the Emergency Department (ED), views of the utility of MAT, and abilities to link patients with opioid use disorder (OUD) to MAT programs in their respective communities.

Methods

This was a cross-sectional survey study of American emergency medicine (EM) physicians with a self-administered online survey via SurveyMonkey (Survey Monkey, San Mateo, California). The survey was emailed to the Council of Residency Directors in Emergency Medicine (CORD) listserv and HCA Healthcare affiliated EM residency programs’ listservs. Attendings and residents of all post-graduate years participated. Questions assessed perceptions of barriers to starting OUD patients on MAT, knowledge of the X-waiver, and knowledge of MAT details. Statistics were performed with JMP software (SAS Institute Inc., Cary, NC) using the two-tailed Z-test for proportions.

Results

There were 98 responses, with 33% female, 55% resident physicians, and an overall 17% response rate. Residents were more eager to start OUD patients on MAT (71% vs 52%, p=0.04) than attendings but were less familiar with the X-waiver (38% vs 73%, p=0.001) or where community outpatient MAT facilities were (21% vs 43%, p=0.02).

Conclusion

Barriers in the ED were identified as a shortage of qualified prescribers, the lengthy X-waiver process, and the poor availability of outpatient MAT resources. EM residents showed more willingness to prescribe MAT but lacked a core understanding of the process. This shows an area of improvement for residency training as well as advocacy among attendings.

## Introduction

The emergency department (ED) continues to exist as a primary avenue through which many patients receive medical care. This is particularly true for patients presenting with recreational drug overdoses and illicit drug-related complications [1]. In 2017, the United States reported just over 70,000 drug overdose deaths, of which 68% involved opioids; this represents an increase of 12% compared to 2016 [2]. Opioids are natural (e.g. morphine or codeine), semi-synthetic (e.g. hydrocodone or oxycodone), or synthetic substances (e.g. fentanyl, tramadol, methadone) that antagonize one of the three main opioid receptor systems (mu, kappa, delta), with the mu receptor being the most involved in addiction physiology. Although opioids can have analgesic effects, they also depress the central nervous system and cause euphoria, giving them a heightened potential for abuse. ED visits and hospitalization utilization for reasons related to opioid use continue to rise, which highlights the severity of this public health crisis [3].

An important component of caring for patients that use illicit drugs includes a discussion about initiating treatment for drug rehabilitation and addiction recovery services. For patients with opioid use disorder (OUD) in particular, medication-assisted treatment (MAT) is the standard of care [4]. Recently, the Substance Abuse and Mental Health Services Administration (SAMHSA) has proposed to replace the traditional term MAT with “medication for opioid use disorder” (MOUD), as MOUD has less stigma. Per the DSM-V, OUD is defined as “a problematic pattern of opioid use leading to clinically significant impairment or distress.” Specifically, in a 12-month period, two of the following must be present: (1) opioids are taken in larger amounts or over a longer period than was prescribed, (2) there is a persistent desire or unsuccessful effort to decrease opioid use, (3) a significant amount of time is spent in obtaining the opioid, using the opioid, and/or recovering from its effects, (4) experiencing a craving to use opioids, (5) recurrent opioid use resulting in failed obligations at work, school, and/or home, (6) continued opioid use despite recurrent social or interpersonal problems, (7) important social, occupational, or recreational activities are negatively affected secondary to opioid use, (8) recurrent opioid use in dangerous situations, (9) continued opioid use despite knowing the substance is causing a persistent physical, social, professional and/or psychological problem, (10) exhibits tolerance, and (11) exhibits withdrawal [5,6].

In OUD, MAT is the use of one of three medications (buprenorphine, naltrexone, or methadone) in combination with psychosocial and/or behavioral therapy to help combat addiction [5]. Buprenorphine is a partial mu-opioid receptor agonist with a long half-life, high potency, and “ceiling effect” for both euphoric sensation and adverse effects [7]. This makes it an optimal treatment alternative for patients with OUD presenting to the ED with opioid withdrawal. Furthermore, the buprenorphine/naloxone (ex. Suboxone®) combination can be taken by patients in any setting; they do not need to attend a daily clinic. However, given its high affinity for the receptor, if patients already have opioids on board, it could precipitate immediate withdrawal and all the complications associated with it (e.g. pulmonary edema, tachycardia, anxiety, nausea, vomiting, diarrhea, and abdominal pain).

Naltrexone is a long-acting, competitive mu-opioid receptor antagonist, available orally and as a monthly extended-release intramuscular injection. Given it can out-compete opioids at the desired receptor, this medication can help prevent relapse. Although adherence and improved mortality for patients with OUD taking naltrexone are not as great as those using opioid-agonist-based MAT, naltrexone is still associated with decreased cravings and decreased rate of relapse, especially when combined with psychotherapy [8,9]. Furthermore, in Russia where there is no opioid-agonist-based MAT, a meta-analysis found that naltrexone demonstrated success in relapse prevention and abstinence stabilization [10].

Methadone is a racemic mixture, with one of its enantiomers having 10× the affinity for the μ-opioid receptor compared to opioids as well as antagonizing the N-methyl-d-aspartate (NMDA) receptor. Unlike buprenorphine, which is a partial μ-opioid receptor agonist, methadone is a full opioid agonist. Methadone is available in liquid and pill formulations, with both reaching maximum plasma concentrations by three hours. Its oral bioavailability is high (> 80%), and it has a potentially long half-life of 7-65 h [11]. However, methadone has well-known toxicities, including but not limited to arrhythmias (prolonged QTc), respiratory depression, and hypoglycemia. Furthermore, it has interactions with multiple other medications, as it is metabolized hepatically by the cytochrome P450 system.

MAT is not a detoxification program, but rather an inclusive, long-term treatment plan after a patient has withdrawn from opioids. In the United States, buprenorphine and naltrexone-based MAT can only be started by a provider with a Drug Enforcement Agency (DEA) license and has undergone training for the Drug Addiction Treatment Act of 2000 (DATA 200) waiver. This is colloquially known as the “X-waiver.” Applications for the X-waiver are made through the SAMHS, which is a branch of the Department of Health and Human Services. At the time of this writing, this was the process, but there is a movement to remove the required training from the X-waiver application process. Typically, office-based physicians apply for the X-waiver, but emergency physicians can obtain an X-waiver to start MAT, too. For methadone-based MAT, patients need to be in an opioid treatment program (OTP) certified by SAMHSA [5].

Although the process of MAT seems complicated and necessitates more initial effort on the part of the physician, the evidence for MAT’s success is overwhelming. A 2018 meta-analysis showed that untreated participants had a higher risk of all-cause mortality and overdose mortality compared with those receiving MAT. In addition, retention in MAT of over one year was associated with a lower mortality rate than that with retention ≤1 year. Thus, authors found improved enrollment and adherence to MAT were crucial in reducing mortality in those with OUD [12].

Furthermore, the success of MAT in reducing mortality in those with OUD was demonstrated not only in the United States but also across the world. In Australia, a state-wide study between 1985 and 2006 with n = 42,676 showed treatment with methadone or buprenorphine reduced mortality by 29% compared with periods without treatment [13]. In Sweden, a longitudinal study using the Kaplan-Meier survival estimate technique found untreated patients with heroin dependency OUD had statistically significantly higher rates of mortality (63 times the rate expected without the disorder) compared to a group of former users in methadone maintenance programs (eight times the rate expected without the disorder) [14].

Although the road to recovery using MAT is challenging, starting patients with OUD on MAT is often the biggest hurdle to their success in battling the disease. As the ED is where many patients with OUD seek care, the ED is a preferred place to start MAT as opposed to referring patients to primary care doctors that many patients will never see or to programs they will not be able to enter for weeks to months. Moreover, initiating MAT in the ED can be lifesaving because there is a 5.5% one-year mortality associated with ED patients who presented with a non-fatal overdose [15]. A randomized control trial (RCT) studying 329 patients with OUD over four years found that ED-initiated buprenorphine treatment compared to brief, stabilizing interventions with outpatient referral significantly increased engagement with addiction treatment, reduced self-reported illicit opioid use, and decreased use of inpatient addiction treatment services [16]. Not only does MAT reduce mortality, but timely MAT improves compliance compared to behavior therapy alone [17]. Given that many patients with OUD frequent the ED, either for drug-related complaints (e.g. overdose and withdrawal) or for regular care (many patients with OUD do not have insurance and thus do not have primary doctors), emergency physicians can play a key role in starting this population on life-saving treatment.

While the practicality and success of utilizing the ED to initiate MAT have been demonstrated in previous works [18,19], large-scale support of and participation with this model is lacking. Medications to start MAT can be difficult to access even for highly motivated individuals, mainly due to a shortage of prescribers and treatment programs [8]. Additionally, the lengthy X-waiver process mandated by the DATA 2000 to prescribe medications such as buprenorphine, Suboxone® (buprenorphine/naloxone), and naltrexone is an established obstacle [20]. For the opioid agonists specifically, despite being successfully utilized by many ED-based treatment programs, the stigma of “replacing one opioid with another” remains a barrier [7]. Evidence-based discussions between providers, administrators, and patients regarding the safety and benefits of these medications are essential to promoting a culture of acceptance and optimizing MAT in the ED. Our investigation sought to understand the factors contributing to the nationwide dearth of ED-based MAT, specifically in teaching settings.

Given the proven efficacy of starting MAT in the ED and low overall participation rates of this practice in our own hospital, we sought to elucidate current views of the disconnect. In this study, we aimed to assess physicians’ perceptions of barriers to starting MAT in the ED, views of the utility of MAT, and the abilities of physicians to link OUD patients to MAT programs in their respective communities. We suspected a variety of reasons for the lack of implementation of MAT in the ED, including but not limited to lack of training, lack of knowledge of the process, a perception that the process was too lengthy or complicated, liability, and concerns that this model would promote further opioid abuse. We also wanted to assess differences in opinions between residents and attending physicians to identify potential teaching points that could enhance resident education.

## Materials and methods

This was a cross-sectional survey study of American emergency medicine physicians affiliated with a residency program via a self-administered online survey using SurveyMonkey (Survey Monkey, San Mateo, CA) (Table 1).

**Table 1:** Survey instrument

Question	Answer Choices
1. Gender	free text
2. Years in practice after medical school	free text
3. Do you know what an X-waiver is?	a. yes
	b. no
4. Would you be interested in obtaining an X-waiver?	a. yes
	b. no
5. Would you be interested in starting patients battling opiate addiction on suboxone, if they were interested?	a. yes
	b. no
6. Do you know where to send a patient to obtain clean needles?	a. yes
	b. no
7. Do you know where to send a patient for HIV/hepatitis testing?	a. yes
	b. no
8. Are you aware of any suboxone clinics in your community?	a. yes
	b. no
9. Have you ever referred a patient to a suboxone clinic?	a. yes
	b. no
10. Do you believe suboxone can help a patient recover from addiction?	a. strongly agree
	b. agree
	c. disagree
	d. strongly disagree
11. Are you aware of community resources for rehabilitation programs?	a. yes
	b. no
12. Do you think it would be helpful to hand out naloxone (Narcan®) to patients battling opiate addiction and also to their families?	a. strongly agree
	b. agree
	c. disagree
	d. strongly disagree
13. Do you think handing out naloxone (Narcan®) condones opiate use?	a. yes
	b. no

Questions were generated de novo by the authors and are not based on an existing model. Recipients of the survey came from the following email mailing lists (listservs): Council of Residency Directors in Emergency Medicine (CORD) and Hospital Corporation of America (HCA) affiliated emergency medicine residency programs across the USA. There was a single email announcement to the entire CORD listserv with no incentives and no reminders. The authors contacted all the HCA emergency medicine residency program directors, who forwarded the survey to all members of their residency (i.e. interns, residents, fellows, and attendings) in a single announcement with no reminders or incentives. Questions consisted of either multiple-choice or yes/no answers to assess perceptions of barriers to starting OUD patients on MAT. Respondents self-reported the number of years in practice out of medical school with values of four years and fewer considered to be residents and five years and greater to be attendings. For our purposes, a facility capable of MAT was one that could provide at a minimum buprenorphine/naloxone (Suboxone®) treatment. Statistics were performed by JMP software (SAS Institute Inc., Cary, NC) with a two-tailed Z-test of proportions. Approval was obtained from HCA Healthcare Institutional Review Board (IRB).

There were no sponsors or funding organizations involved in our research. There were no prizes, gifts, or compensation for those who answered the survey.

## Results

A total of 98 physicians out of 576 responded to the survey, with 33% female, 55% resident physicians, and an overall 17% response rate. An overwhelming majority, 80% of respondents, demonstrated an interest in starting OUD patients on MAT, such as buprenorphine/naloxone (Suboxone®), and 94% either “agreed” or “strongly agreed” that MAT was helpful for OUD patients to overcome addiction. However, only 53% had knowledge of the X-waiver (mandated training to prescribe buprenorphine/naloxone [Suboxone®] long-term with the federal DEA), and only 32% knew of an outpatient community facility capable of continuing buprenorphine/naloxone (Suboxone®) management.

**Figure 1:**
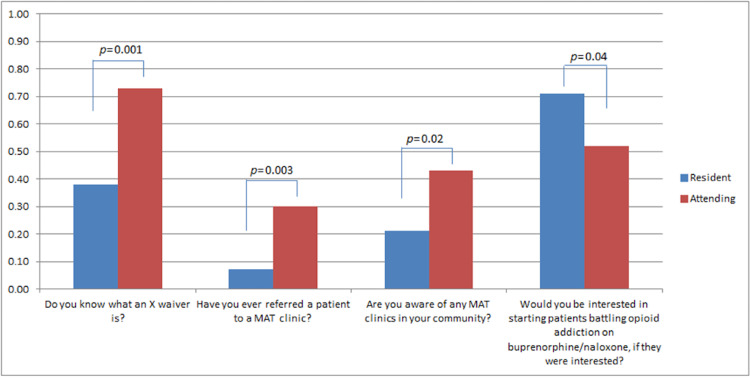
Responses to MAT survey comparing attendings and residents MAT - medication-assisted treatment

When dividing responses by the level of training, residents were more eager to start OUD patients on MAT (71% vs 52%, p=0.04) than attendings but were less familiar with what the X-waiver was (38% vs 73%, p=0.001), where community outpatient MAT facilities were (21% vs 43%, p=0.02), or having ever referred OUD patients to MAT programs in their individual practice (7% vs 30%, p=0.003) (Figure 1).

There was no distinction between post-graduate years in the responses; the survey was open to all emergency medicine residents in an attempt to represent the entire population of nascent doctors. There were no differences between residents and attendings in the perception of the utility of MAT for OUD patients (96% vs 90%, p=0.27) or perception of condoning continued opiate use by prescribing naloxone (Narcan®) to discharged OUD patients from the ED (14% vs 15%, p=0.85). In comparing female vs male respondents, the only significant difference was seen in whether giving naloxone condoned opiate use (6% female vs 20% male, p=0.02). In other words, males were more likely to think that prescribing naloxone condoned opiate use. Otherwise, answers to questions were not statistically significant between males and females.

## Discussion

With the opioid epidemic highlighted by multiple professional medical societies and mainstream media, emergency medicine physicians are keenly aware of the suffering from OUD. The goal of this survey was to assess any physician perceived barriers and current practice experience with starting and referring OUD patients to MAT. Overwhelming support was seen among all levels of emergency medicine physicians to start OUD patients on MAT, which represents an important cultural shift in how we approach this vulnerable patient population. There were no differences between female and male respondents (overall and within training levels), except when all females were less likely to think that giving naloxone condones opiate use compared to all males (p < 0.05). This suggests the differences in opinions between attendings and residents is not attributable to gender differences but rather differences in the prevailing thoughts on OUD from training in medical schools and residencies at different points in time.

The data further suggest the need for more robust residency training about OUD, MAT, the X-waiver process, and the ED’s role in the timely bridging of patients with OUD to gold-standard treatment. A willingness to start OUD patients on MAT was greater in resident-level physicians compared to attendings, but the knowledge of prescribing details and referring to qualified centers fell behind that of attendings. Residency training can consider including X-waiver requirements prior to starting residency or include it as part of first-month onboarding. Resident-run initiatives should also be encouraged to capitalize on their enthusiasm to treat OUD patients. Additionally, teaching faculty and residency program leadership should model behaviors that encourage residents to continue MAT prescribing in their practices after graduation. It is unclear based on our survey why attending physicians overall were more hesitant. A follow-up survey study can be used to further investigate this significant pattern.

At the time of this writing, those wishing to obtain an X-waiver had to apply through SAMSHA and complete training. However, there was speculation about the training being removed for those physicians treating less than 30 patients. This likely would not affect emergency medicine residents or attendings, as both groups see more than 30 patients with OUD per year. Furthermore, this survey was administered based upon the process at the time, which mandated the training.

Unfortunately, the vital, widespread support from hospital and health system administrations, group leaderships, and insurance policies to efficiently enact meaningful changes in the ED approach to OUD patients were not assessed. However, the views and abilities of emergency physicians to start MAT remains an important component of that system. The X-waiver itself is a barrier to initiating effective MAT and becoming waivered will remain an integral part of helping OUD patients until laws can be changed to ease the requirement. At the time of this writing, there was an effort on behalf of professional medical organizations such as the American College of Emergency Physicians (ACEP) and the American Medical Association (AMA) at the federal level attempting to remove the X-waiver process and at the very least streamline it. Available outpatient clinics that continue MAT are independent of emergency medicine physicians’ abilities and scope of practice, but maintaining close relationships with these facilities and widespread distribution of information on available resources is critical to MAT’s success. Many EDs have had success in starting their own ED-based MAT clinics but have required extensive hospital support [18].

Given these obstacles, patients with OUD desperate to wean themselves from opioids or treat their opioid dependence may turn to alternative agents. These agents include using prescription medications, such as clonidine or gabapentin, in an off-label capacity or over-the-counter medications, such as loperamide, in supratherapeutic doses. Finally, individuals may use herbal supplements like kratom to prevent opioid withdrawal, which is increasingly dangerous as the Food and Drug Administration (FDA) does not regulate these supplements. Each of these therapies for OUD is associated with unique toxicities that providers should recognize, as patients will likely minimalize in their attempt to self-treat [11].

Further research is needed across non-HCA Healthcare emergency medicine residencies and in EDs that do not have a residency program to fully represent emergency physicians’ views. Additionally, with the advancement of emergency department ARNPs and PAs, they should also be surveyed regarding their views of OUD, MAT, and the X-waiver. Finally, there are different barriers and stigma throughout the country, so it would be prudent to further delineate views by region and have targeted education for each of those regions to reduce their specific bias.

Limitations

A major limitation of our study was a response rate of only 17% of the listservs members surveyed. Given the small sample size, it might be difficult to extrapolate our findings to residents and attendings nationwide secondary to sample bias. We neither performed a non-responder survey nor analyzed the demographics of those who did not respond.

Another limitation is the population of emergency medicine residents. The residency classes that were surveyed were all HCA-affiliated emergency medicine residency programs, which are primarily located in the Atlantic South region. Thus, our sample may not represent practice patterns for all residency programs across the country. Many of the emergency medicine residencies are at hybrid community hospitals as well as tertiary urban centers. Furthermore, these EDs are all under the direction of HCA Healthcare, which has its own approval processes, and not all EDs have MAT medications like methadone, naltrexone, and buprenorphine available in the ED even if the emergency physicians had obtained an X-waiver. The organization is aware of the need for MAT and MAT-trained providers, which is why they started four addiction medicine fellowships in 2020 and plan to add even more over the coming years [21]. However, it is not clear if these fellows will be able to offer ED-based MAT.

The CORD listserv included a greater geographic representation of attendings but by definition are not solely community-based practicing attendings. Therefore, the CORD responses may not represent the population of community-based providers.

Despite the limitations, our results could be arguably extrapolated to HCA Healthcare emergency departments in the Atlantic South that, according to the 2020 US Census, has over a third of the US population, which has grown compared to previous Census years [22]. This implies that, although our sample residents are largely from one area of the country, these are the providers that are and will be caring for a large portion of the nation’s patients.

## Conclusions

In summary, support for MAT is robust among all levels of emergency medicine physicians but knowledge of community resources and required training (like the X-waiver) to prescribe certain forms of MAT remains a barrier. When comparing resident physicians to attending physicians, the knowledge gap of community MAT facilities and the practice of referring OUD patients for MAT is wide, demonstrating clear areas of residency training to bolster. Additionally, resident physicians expressed more willingness to initiate MAT for patients with OUD, and therefore allowing resident-run initiatives during residency program could encourage quicker adaptation of MAT prescribing in the ED. Finally, perhaps more MAT advocacy is needed for attendings. The ED is increasingly becoming the starting point to propel patients with OUD into appropriate MAT programs and emergency medicine physicians are willing and eager to help.

## Appendices

**Table 2:** Survey Reponses Data

			At which program are you currently working/affiliated?															Do you know what an X waiver is?		Would you be interested in obtaining an X waiver?		Would you be interested in starting patients battling opiate addiction on Suboxone®, if they were interested?		Do you know where to send a patient to obtain clean needles?		Do you know where to send a patient for HIV/hepatitis testing?		Are you aware of any Suboxone® clinics in your community?		Have you ever referred a patient to a Suboxone® clinic?		Do you believe Suboxone® can help a patient recover from addiction?				Are you aware of community resources for rehabilitation programs?		Do you think it would be helpful to hand out naloxone (Narcan®) to patients battling opiate addiction and also to their families?				Do you think handing out naloxone (Narcan®) condones opiate use?	
Female	Male	Years in practice after medical school:	Aventura Hospital and Medical Center	Oak Hill Hospital	North Florida Regional Medical Center (Gainesville)	Kendall Regional Medical Center	Ocala Regional Medical Center	Osceola Regional Medical Center	Coliseum Medical Center	Orange Park Medical Center	Brandon Regional Hospital	MountainView Hospital- Sunrise Health	Grand Strand Regional Medical Center	Riverside Community Hospital/ University of California Riverside	St. Lucie Medical Center	Council of Emergency Medicine Residency Director List Serv	Other (please specify)	Yes	No	Yes	No	Yes	No	Yes	No	Yes	No	Yes	No	Yes	No	Strongly agree	Agree	Disagree	Strongly disagree	Yes	No	Strongly agree	Agree	Disagree	Strongly disagree	Yes	No
Female		1					X												No	Yes		Yes			No	Yes			No		No		Agree			Yes			Agree				No
	Male	2				X													No		No		No		No		No		No		No		Agree				No	Strongly agree					No
	Male	2					X												No		No	Yes			No	Yes			No		No		Agree			Yes			Agree				No
	Male	1							X									Yes		Yes		Yes			No	Yes			No		No		Agree			Yes			Agree				No
Female		2				X													No	Yes		Yes			No	Yes			No		No		Agree				No		Agree				No
	Male	2				X													No	Yes		Yes			No		No		No		No		Agree				No	Strongly agree					No
Female		1					X												No		No	Yes			No	Yes			No		No	Strongly agree					No		Agree			Yes	
	Male	12			X													Yes			No		No		No	Yes			No		No		Agree			Yes		Strongly agree					No
	Male	20			X														No		No	Yes			No	Yes		Yes			No		Agree			Yes			Agree				No
	Male	1			X													Yes		Yes		Yes			No		No		No		No		Agree				No	Strongly agree					No
	Male	3					X												No	Yes		Yes			No	Yes			No		No	Strongly agree					No	Strongly agree					No
	Male	1							X										No	Yes		Yes			No	Yes			No		No		Agree			Yes			Agree				No
Female		1							X										No	Yes		Yes			No	Yes			No		No	Strongly agree					No	Strongly agree					No
	Male	1							X									Yes		Yes		Yes			No		No		No		No	Strongly agree				Yes			Agree				No
																												Yes		Yes			Agree				No	Strongly agree					No
	Male	1							X										No	Yes		Yes			No		No		No		No		Agree			Yes			Agree			Yes	
	Male	22							X									Yes		Yes		Yes			No	Yes		Yes			No		Agree			Yes			Agree			Yes	
	Male	25						X											No		No	Yes			No	Yes			No		No	Strongly agree					No		Agree			Yes	
	Male	2															Franciscan-Midwestern University Chicago		No	Yes		Yes			No	Yes		Yes			No			Disagree		Yes		Strongly agree					No
	Male	9															TTUHSC El Paso	Yes		Yes		Yes			No	Yes			No		No	Strongly agree				Yes			Agree				No
	Male	3															Midwestern/St James Emergency Medicine Residency		No		No		No		No	Yes			No		No		Agree			Yes			Agree				No
	Male	26															TTUHSC El Paso		No		No		No		No	Yes			No		No		Agree			Yes			Agree				No
	Male	2										X							No	Yes		Yes		Yes			No		No		No		Agree			Yes		Strongly agree					No
	Male	5															Texas Tech University Medical Center		No		No	Yes			No	Yes			No		No		Agree			Yes				Disagree		Yes	
	Male	9															Midwestern University/CCOM EM	Yes			No		No		No	Yes		Yes		Yes			Agree			Yes		Strongly agree					No
	Male	6															Franciscan Health Olympia Fields Emergency Medicine	Yes		Yes		Yes		Yes		Yes			No		No		Agree			Yes			Agree			Yes	
Female		2	X																No	Yes		Yes			No	Yes			No		No		Agree			Yes			Agree				No
Female		3	X															Yes		Yes		Yes			No		No		No		No	Strongly agree				Yes		Strongly agree					No
	Male	11														X		Yes		Yes		Yes			No	Yes			No		No		Agree			Yes			Agree				No
	Male	3															Franciscan Health Olympia Fields Emergency Medicine		No		No	Yes			No	Yes			No		No	Strongly agree					No	Strongly agree					No
Female		13														X		Yes		Yes		Yes			No		No		No		No		Agree			Yes		Strongly agree					No
Female		1															Jefferson Health Northeast		No		No	Yes			No		No	Yes			No	Strongly agree				Yes		Strongly agree					No
	Male	5															TTUHSC El Paso	Yes		Yes		Yes		Yes		Yes		Yes		Yes		Strongly agree				Yes		Strongly agree					No
Female		4						X										Yes		Yes		Yes			No	Yes			No		No		Agree				No		Agree				No
Female		9						X											No		No		No		No	Yes		Yes		Yes				Disagree		Yes			Agree				No
Female		2															Jefferson Health Northeast	Yes		Yes			No	Yes		Yes		Yes			No	Strongly agree				Yes		Strongly agree					No
	Male	3						X											No	Yes		Yes			No		No	Yes			No	Strongly agree					No	Strongly agree					No
	Male	1						X										Yes			No		No	Yes		Yes		Yes			No		Agree			Yes			Agree				No
Female		3															Jefferson Health Northeast		No		No	Yes		Yes		Yes			No		No	Strongly agree				Yes		Strongly agree					No
Female		3						X											No	Yes		Yes			No	Yes			No		No	Strongly agree					No	Strongly agree					No
		3															Jefferson Health Northeast		No		No		No		No	Yes			No		No	Strongly agree				Yes		Strongly agree					No
	Male	16															Jefferson Health Northeast	Yes			No		No		No	Yes			No	Yes				Disagree		Yes			Agree				No
	Male	4															Jefferson Health Northeast	Yes		Yes		Yes		Yes		Yes		Yes		Yes		Strongly agree				Yes			Agree			Yes	
Female		8															Jefferson NE Torresdale	Yes		Yes		Yes		Yes		Yes			No		No		Agree			Yes			Agree				No
	Male	3															Texas Tech University Medical Center	Yes		Yes		Yes			No		No		No		No	Strongly agree					No				Strongly disagree	Yes	
	Male	18															Jefferson Health Northeast	Yes		Yes		Yes			No	Yes		Yes		Yes		Strongly agree				Yes		Strongly agree					No
	Male	1															TTUHSC El Paso		No	Yes		Yes			No		No		No		No		Agree			Yes			Agree			Yes	
	Male	13															UMKC- Truman Medical Center	Yes		Yes		Yes			No	Yes		Yes		Yes		Strongly agree				Yes		Strongly agree					No
	Male	25															TTUHSC El Paso	Yes		Yes		Yes		Yes		Yes		Yes		Yes			Agree			Yes		Strongly agree					No
	Male	5															TTUHSC El Paso		No	Yes			No		No	Yes			No		No		Agree			Yes			Agree				No
	Male	6															TTUHSC El Paso	Yes			No	Yes			No	Yes			No		No	Strongly agree				Yes			Agree			Yes	
	Male	1															TTUHSC El Paso	Yes		Yes		Yes			No	Yes		Yes			No		Agree				No		Agree				No
	Male	2															TTUHSC El Paso	Yes		Yes		Yes			No		No		No		No	Strongly agree				Yes		Strongly agree					No
	Male	22															TTUHSC El Paso	Yes			No		No		No	Yes		Yes			No		Agree			Yes				Disagree		Yes	
Female		12														X		Yes		Yes		Yes		Yes		Yes		Yes		Yes		Strongly agree				Yes		Strongly agree					No
	Male	11															TTUHSC El Paso		No		No	Yes			No	Yes			No		No		Agree			Yes			Agree				No
Female		2.5															TTUHSC El Paso	Yes		Yes		Yes			No	Yes		Yes		Yes		Strongly agree				Yes		Strongly agree					No
	Male	13															TTUHSC El Paso	Yes		Yes		Yes			No		No		No		No	Strongly agree				Yes			Agree				No
	Male	38														X		Yes		Yes		Yes			No		No	Yes			No	Strongly agree				Yes			Agree				No
	Male	9															TTUHSC El Paso	Yes		Yes		Yes			No	Yes			No		No		Agree			Yes			Agree				No
Female		24															TTUHSC El Paso		No		No	Yes			No	Yes			No		No		Agree			Yes			Agree				No
	Male	19															LewisGale Medical Center		No		No		No		No	Yes			No		No			Disagree		Yes			Agree				No
Female		11														X		Yes			No	Yes			No	Yes			No		No		Agree			Yes		Strongly agree					No
Female		10														X		Yes			No		No		No	Yes			No		No		Agree			Yes		Strongly agree					No
	Male	14														X		Yes		Yes		Yes		Yes		Yes		Yes		Yes		Strongly agree				Yes		Strongly agree					No
	Male	13															Thomas Jefferson University	Yes		Yes		Yes			No		No	Yes		Yes			Agree				No	Strongly agree					No
Female		1			X													Yes			No	Yes			No	Yes			No		No		Agree			Yes		Strongly agree					No
Female		2						X											No	Yes		Yes			No	Yes			No		No	Strongly agree					No	Strongly agree					No
Female		2									X								No		No	Yes			No		No	Yes		Yes			Agree			Yes			Agree			Yes	
	Male	1									X							Yes		Yes		Yes			No	Yes			No		No		Agree			Yes			Agree				No
	Male	3						X											No	Yes		Yes			No	Yes			No		No		Agree			Yes			Agree			Yes	
	Male	1						X											No	Yes		Yes			No		No		No		No		Agree			Yes			Agree				No
	Male	1						X										Yes		Yes		Yes			No	Yes			No		No	Strongly agree				Yes		Strongly agree					No
Female		2						X										Yes		Yes		Yes			No		No	Yes		Yes		Strongly agree					No	Strongly agree					No
	Male	3						X											No	Yes		Yes			No	Yes			No		No	Strongly agree				Yes			Agree				No
	Male	2						X											No	Yes		Yes			No	Yes			No		No		Agree			Yes			Agree				No
Female		3						X											No	Yes		Yes			No	Yes			No		No				Strongly disagree	Yes			Agree				No
Female		17												X				Yes			No	Yes			No		No	Yes		Yes		Strongly agree				Yes		Strongly agree					No
	Male	2						X											No	Yes			No		No	Yes			No		No		Agree			Yes			Agree				No
Female		3												X					No	Yes		Yes			No		No		No		No	Strongly agree				Yes		Strongly agree					No
	Male	1												X					No		No	Yes			No	Yes			No		No		Agree				No	Strongly agree					No
	Male	2										X						Yes		Yes		Yes		Yes			No		No		No	Strongly agree				Yes		Strongly agree					No
	Male	21												X					No		No	Yes			No	Yes			No		No		Agree			Yes			Agree				No
	Male	28												X				Yes		Yes		Yes			No	Yes		Yes		Yes			Agree			Yes		Strongly agree					No
	Male	11												X				Yes		Yes		Yes			No	Yes		Yes			No		Agree			Yes		Strongly agree					No
	Male	1												X					No		No	Yes			No	Yes			No		No		Agree			Yes			Agree				No
	Male	2										X						Yes			No		No		No	Yes			No		No		Agree			Yes				Disagree		Yes	
Female		19						X										Yes			No		No		No		No	Yes			No			Disagree		Yes			Agree				No
	Male	2									X							Yes			No	Yes			No	Yes		Yes			No		Agree			Yes			Agree			Yes	
Female		2						X										Yes		Yes		Yes			No	Yes		Yes			No		Agree			Yes			Agree				No
	Male	19										X							No	Yes			No		No	Yes			No		No		Agree			Yes				Disagree			No
Female		23						X										Yes			No		No		No	Yes		Yes			No		Agree			Yes			Agree				No
	Male	2					X												No	Yes		Yes			No	Yes			No		No	Strongly agree				Yes		Strongly agree					No
Female		2			X													Yes		Yes		Yes			No	Yes			No		No		Agree				No	Strongly agree					No
	Male	15			X													Yes			No		No		No	Yes			No		No		Agree			Yes		Strongly agree					No
	Male	1						X											No		No	Yes			No	Yes			No		No		Agree			Yes			Agree				No
Female		10						X										Yes		Yes		Yes			No	Yes			No		No		Agree			Yes		Strongly agree					No
Female		2						X										Yes		Yes		Yes			No	Yes			No		No	Strongly agree					No	Strongly agree					No
Female		8						X											No		No	Yes			No	Yes			No		No		Agree			Yes			Agree				No
	Male	1																Yes		Yes		Yes			No	Yes		Yes		Yes		Strongly agree				Yes		Strongly agree					No
Total female: 33	Total female: 65	Average years out of medical school: 7.4	2	0	6	3	5	22	6		3	4	0	7	0	7	33																										
33.30%	66.30%	Median years out of medical school: 3																53% yes	46% no	62.6% yes	37.3% no	79.7% yes	20% no	12% yes	87.8% no	76.7% yes	23.2% no	32% yes	68% no	18% yes	82% no	36% strongly agree	58% Agree	5% Disagree	1% Strongly disagree	79% yes	21% no	46% Strongly agree	49% Agree	4% Disagree	1% Strongly diagree	15% yes	85% no
